# Abrasion Wear Resistance of Precipitation-Hardened Al-Zn-Mg Alloy

**DOI:** 10.3390/ma17102446

**Published:** 2024-05-19

**Authors:** Tomislav Rodinger, Helena Lukšić, Danko Ćorić, Vera Rede

**Affiliations:** Department of Materials, Faculty of Mechanical Engineering and Naval Architecture, University of Zagreb, Ivana Lučića 5, 10000 Zagreb, Croatia; tomislav.rodinger@fsb.unizg.hr (T.R.); helena.luksic@fsb.unizg.hr (H.L.); vera.rede@fsb.unizg.hr (V.R.)

**Keywords:** aluminum alloy, precipitation hardening, abrasion, wear rate, critical abrasive grain size

## Abstract

The heat treatment of aluminum alloys is very important in industries where low weight in combination with high wear resistance, good strength, and hardness are important. However, depending on their chemical composition, aluminum alloys are subjected to different mechanical and thermal treatments to achieve the most favorable properties. In this study, an Al-Zn-Mg alloy was heat-treated including solution annealing at 490 °C for 1 h with subsequent artificial aging at 130, 160, and 190 °C for 1, 5, and 9 h. The hardness (HV1) and abrasive wear resistance with three different abrasive grain sizes were measured for all samples. The highest hardness was measured for the samples artificially aged at 130 °C/5 h, 227 HV1, while the lowest hardness was measured for the samples aged at 190 °C/9 h. The highest and the lowest wear resistance was also observed for the same state, i.e., artificially aged at 130 °C/5 h and 190 °C/9 h, respectively. The critical abrasive grain size was detected for some samples, where a decrease in wear rate was observed with an increase in the abrasive grain size from the medium value to the largest. The Response Surface Methodology (RSM) was applied to demonstrate the influence of the input parameters on the material wear rate.

## 1. Introduction

The development of aluminum alloys is very important from the perspective of light-weight engineering, mainly in aeronautical [1,2] and automotive industries [3,4], where it can reduce up to 60% of the mass compared to the steel part it replaces [5,6]. In the case where a high strength–weight ratio is needed, aluminum alloy properties often need to be improved. This can be achieved by alloying with different elements and varying their content, cold working, or heat treatment.

Heat-treatable Al alloys are mostly solution-treated at temperatures above 450 °C to dissolve alloying elements in the aluminum matrix. Solution treatment is followed by quenching to obtain a supersaturated solid solution (SSS) α-Al matrix. Then, the material is aged at room (natural aging) or higher temperature (artificial aging) [7]. The increase in mechanical properties is the result of the formation of precipitates from the SSS α-Al matrix [8].

Al-Zn-Mg alloys are generally characterized by especially large yield strength and ultimate tensile strength (UTS) after aging [9]. In these alloys, magnesium is the main alloying element that increases the strength by forming the *η* (MgZn_2_) intermetallic phase, which has a high solid solubility in the Al matrix [10], according to the following equation [11,12]:

α-Al SSS → Guinier-Preston (GP) zones → *η*′ (MgZn_2_) → *η* (MgZn_2_). (1)

GP zones are nanosized coherent pre-precipitates (or solute clusters) that are formed during natural aging and in the early stages of artificial aging from which the semi-coherent transitional metastable *η*′ phase, and eventually, the final incoherent equilibrium *η* phase nucleate and grow [12]. With the increase in Mg content, the volume fraction of *η* precipitates increases, which improves the yield strength, but lowers toughness and promotes the probability of intergranular fracture [13,14]. Zinc promotes the nucleation and growth of precipitates during age hardening, which also increases the strength of the alloy and shortens the heat treatment time [10].

A very large number of problems in industry are caused by abrasive wear, and it is assumed that this results in costs in the amount of 1–4% of the gross national product of industrialized countries [15]. To minimize these costs, great efforts are being made to investigate how to increase the wear resistance of various structural materials. In the scope of the 7xxx group of Al alloys, Reis et al. [16] investigated abrasive wear of two different Al-Zn-Mg alloys and proposed a correlation between wear, hardness, and UTS, where wear decreases with the increase in hardness and UTS. Focusing on different Zn and Mg content in Al-Zn-Mg alloys, Pruthvi and Shenoy [17] investigated wear properties after solution and retrogression re-aging heat treatment. Their study concludes that the wear rate decreases, i.e., the material is more wear-resistant with the increase in Zn content and re-aging duration. With the increase in aging temperature and time, the size of the precipitates increases, but this does not mean that the wear rate increases. Yildirim et al. [18] found that 7075 Al alloy has the lowest wear rate when aged at 120 °C for 24 h, even though MgZn_2_ precipitates continue to grow with higher temperatures or longer aging times.

**Figure 1:**
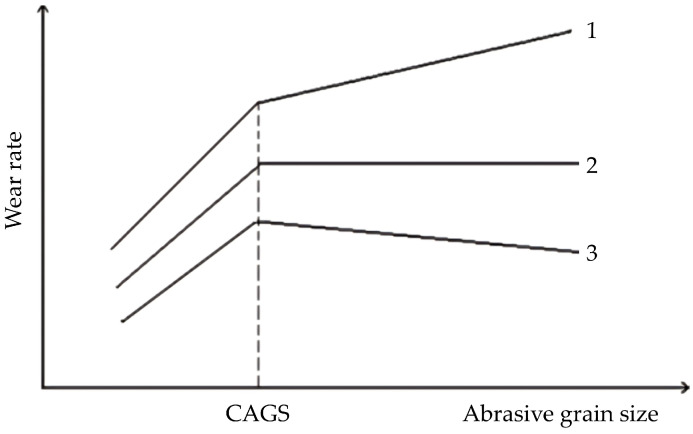
Types of wear rate curves depending on the abrasive grain size where CAGS has been noticed [19].

The influence of the abrasive grain size on the wear rate has already been studied, and in some cases, the critical abrasive grain size (CAGS) has been noticed, as shown in Figure 1. In those studies, three main types of curves have been described: after reaching the CAGS, the wear rate continues to increase but with lower intensity (curve 1), or the wear rate remains unchanged (curve 2), while in some cases it even starts to decrease (curve 3) [19].

This phenomenon of the change in wear rate trend with varying abrasive size can be observed in various metallic [19,20,21,22] and non-metallic [23] materials.

By the application of the design of experiment (DOE) method and statistical analysis of the obtained results, the optimal regime of artificial aging which resulted in the lowest wear rate was determined. The experimental results were statistically studied by the analysis of variance (ANOVA) and represented by the Response Surface Methodology (RSM). These methods are widely accepted in different areas, especially in materials research. They were used to optimize the sintering parameters of alumina ceramics [24], to evaluate boride surface layer thickness on steel [25], tool wear during the machining of Al/SiC composite [26], but also to compare the aging parameters of Al alloys on their yield strength, hardness [27], and thermal properties [28].

This research aimed to investigate the influence of different artificial aging parameters (temperature and time) of Al-Zn-Mg alloy on its abrasive wear rate by different abrasive grain sizes concerning the hardness achieved.

## 2. Materials and Methods

For the intended experiments, an Al alloy was used whose chemical composition is shown in Table 1. The analysis of the chemical composition was performed with an optical emission spectrometer GDS 850A (Leco, Saint Joseph, MI, USA). Based on the chemical composition, it is clear that this is an Al-Zn-Mg alloy. These aluminum alloys from the 7xxx series are known for their high specific strength, stiffness, processability, and weldability, and are widely used in the aerospace, rail traffic, and civil infrastructure industries [29,30,31].

**Figure 2:**
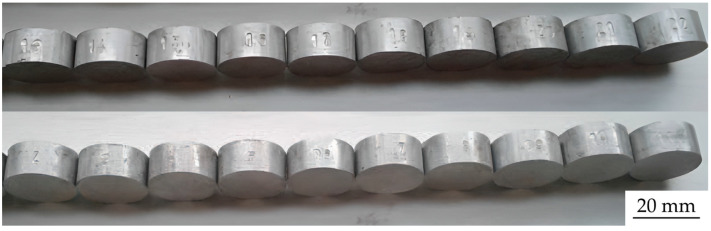
Two groups of Al samples.

A total of two groups with 10 samples each were cut from the Al rod using a band saw with water cooling. The samples were cylindrical with a diameter of 20 mm and a height of 10 mm, as shown in Figure 2.

After analyzing the chemical composition, the density of the material was measured which was later used for the determination of volume loss (Δ*V*) and wear rate (*ώ*) during abrasion tests. Density was measured by the Archimedes principle with an analytical balance type JP703C (Mettler Toledo, Zürich, Switzerland). The mean density value of the three measurements was *ρ* = 2.7857 g/cm^3^.

After determining the chemical composition, the heat treatment temperatures were chosen. Precipitation hardening was carried out in an electric furnace (Over, Sveta Nedelja, Croatia). Solution treatment of all samples was performed at 490 °C for 1 h, followed by quenching in water. The temperature for solution treatment was determined based on the ternary Al-Zn-Mg diagram available in [32]. One sample (ST-Q) was only solution-treated and quenched in water, without subsequent artificial aging, while the other samples were subjected to artificial aging. The temperatures and times of artificial aging were varied on three levels (130, 160, and 190 °C; 1, 5, and 9 h, respectively) as shown in Table 2. Two samples were subjected to the same aging regime.

**Figure 3:**
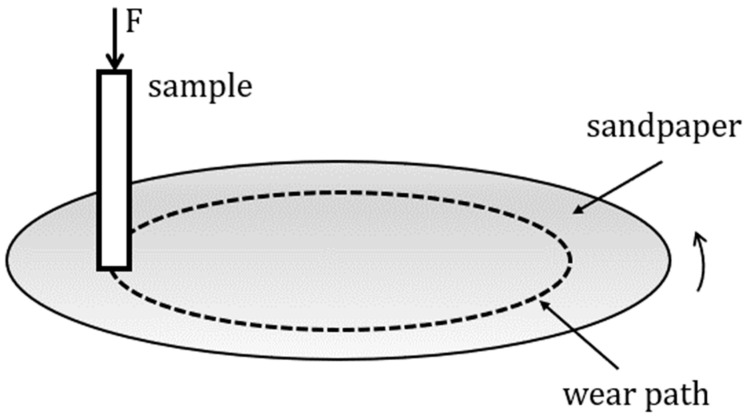
Schematic representation of tests with the Taber abraser device [33].

After heat treatment, the samples were tested for wear resistance with the Taber abraser device (Taber Industries, North Tonawanda, NY, USA) with a rotating abrasive disc of 125 mm in diameter. The wear test samples were cut into the shape of a four-sided prism with dimensions of approximately 5 mm × 5 mm × 10 mm, and the cross-sectional area of 5 mm × 5 mm was worn. From each cylindrical sample, one prism for the wear test was cut so that each thermal state was tested on two samples. During the test, the samples were pressed against a rotating disk with mounted sandpaper with a constant force of 4.91 N. The disk rotated at a speed of 60 rpm, and the relative tangential speed of the sample was 0.251 m/s. Figure 3 schematically shows the testing technique. For the abrasion wear resistance, three types of Al_2_O_3_ sandpaper with different grain sizes were used, as shown in Table 3. The samples were tested for 100 s on each sandpaper, which corresponds to an approximate wear length of 25 m. The wear tests were carried out with sandpapers in the following order: P600, P280, and P180. All the samples were in about the same initial condition before each test, i.e., they had similar roughness of the test surface. The tested surface of the samples was polished with sandpaper P800 before each wear test to ensure approximately the same surface quality. This eliminated the stress concentrator effect determined by the degree of roughness, which can ultimately be reflected in the hardness values of the surface layer. Before and after the wear test, the sample mass was measured on a precision balance with a resolution of 0.0001 g (type B5C 1000, Mettler Toledo, Zurich, Switzerland) to determine the mass loss (Δ*m*) and convert it to volume loss (Δ*V*). Also, before each wear test, the cross-sectional area of the worn surface was measured to calculate the abrasive wear rate (*ώ*) as a ratio of the volume loss and wear surface.

**Figure 4:**
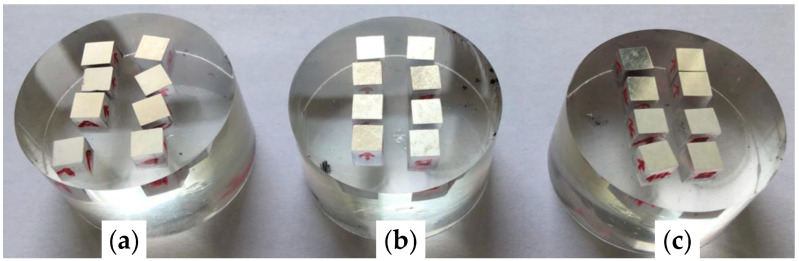
Metallographically prepared samples aged at (**a**) 130 °C; (**b**) 160 °C; (**c**) 190 °C.

The hardness of the samples was measured after metallographic preparation, which included mounting, planar grinding, rough polishing, and final polishing, as shown in Figure 4. The grinding of the samples was carried out in four steps using sandpapers of different sizes of SiC abrasive particles on the device Phoenix Alpha (Buehler, Lake Bluff, IL, USA). The sandpapers were used in the following order: P600, P1200, P2500, and P4000. The grinding plate rotated at a speed of 300 rpm. During the grinding process, the samples were cooled with water to avoid possible microstructural transformations due to heating. After that, a two-step polishing process was carried out on the same device. As a polishing base, a cloth with diamond paste applied was used. The cooling agent during the polishing was a lubricant. In the first step, diamond paste with a 3 µm average diameter of abrasive particles was used, while a polishing liquid with a 0.03 µm average diameter of abrasive particles was used in the second polishing step. During the polishing, the plate rotated at a speed of 150 rpm. The hardness measurement was carried out by the standard Vickers method with the Indentec device (ZwickRoell, Ulm, Germany), applying an indentation load of 9.81 N (HV1). Ten hardness measurements were performed on each sample and the mean value was calculated.

For the applicability of the Response Surface Methodology (RSM) based on the obtained data, the results were also processed statistically. For the design of experiment (DOE) and analysis of the obtained results, Design-Expert 13.0.5 software (Stat-Ease, Minneapolis, MN, USA) was used. DOE had three independent variables: artificial aging temperature (A), artificial aging time (B), and average abrasive grain size (C) with three levels of variations: low (−1), center (0), and high (+1), as shown in Table 4. As the real average abrasive grain size for sandpaper quality P280 is not exactly the in the middle of grain sizes for P600 and P180, but it is very close, the center level for abrasive grain size was set as 53.9 µm, which is the average value of the largest and the smallest abrasive grains used.

## 3. Results and Discussion

**Figure 5:**
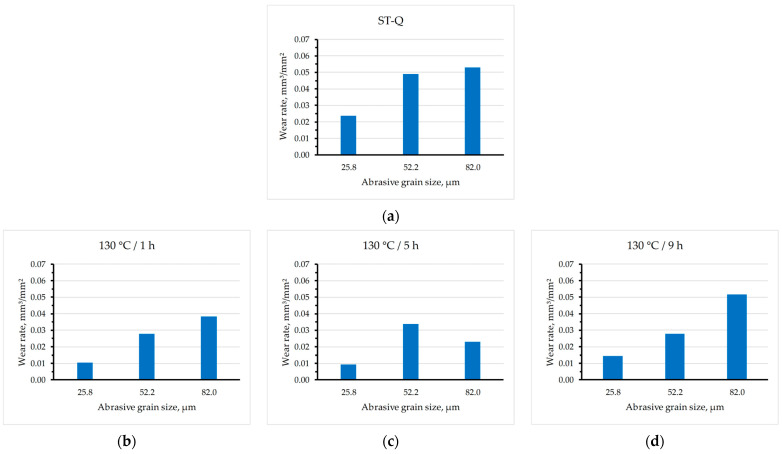

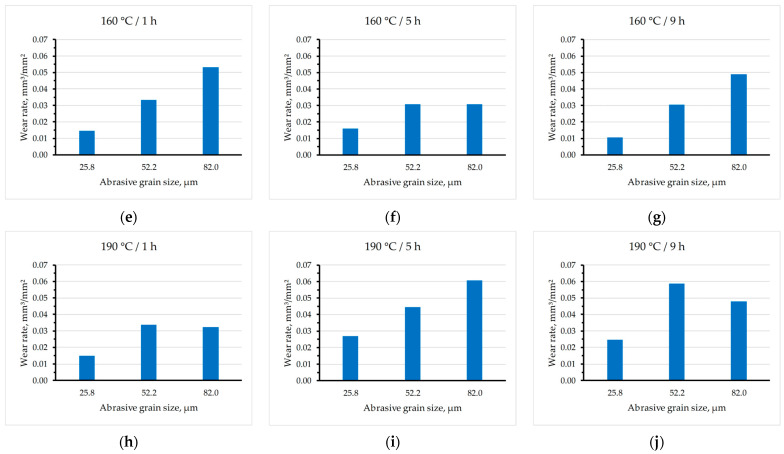
Wear rate of states: (**a**) ST-Q; (**b**) 130/1; (**c**) 130/5; (**d**) 130/9; (**e**) 160/1; (**f**) 160/5; (**g**) 160/9; (**h**) 190/1; (**i**) 190/5; (**j**) 190/9; abraded with different abrasive grain sizes.

Table 5 shows the mean values of mass loss (with standard deviations) and the total average wear rates after abrasion with different sandpaper qualities. The abrasion was tested by three different grits of sandpaper (P600, P280, and P180) for 100 grinding cycles. The samples aged at 130 °C for 5 h showed the lowest total wear rate (the highest wear resistance), and the samples aged at 190 °C for 5 and 9 h had the highest total wear rates (the lowest wear resistance). In Figure 5, the wear rate is represented as a function of the abrasive grain size for each state.

For some thermal states, the wear rate increases linearly with increasing abrasive grain size (130/1, 160/1, 160/9, and 190/5) or even exponentially (130/9), and remains approximately constant for the last two abrasive sizes (160/5 and 190/1), or begins to decrease (130/5 and 190/9). The phenomenon of critical abrasive grain size is visible for the states ST-Q, 130/5, 130/9, 160/5, 190/1, and 190/9, while at other states (130/1, 160/1, 160/9, and 190/5) it is not observed.

**Figure 6:**
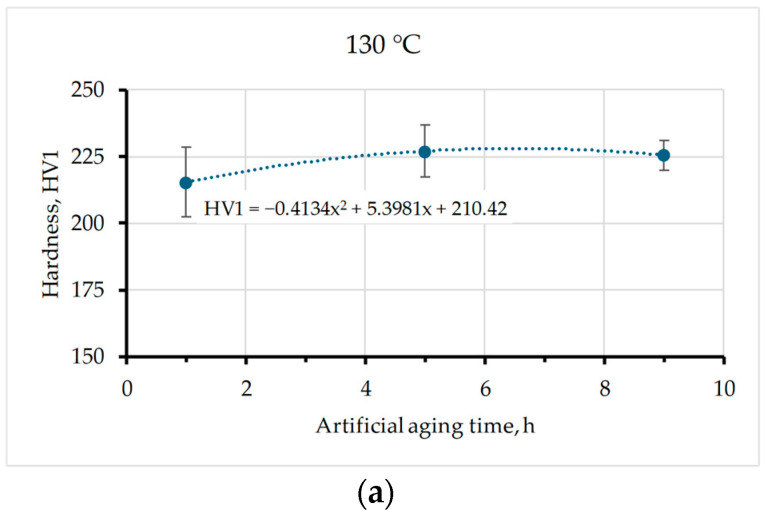

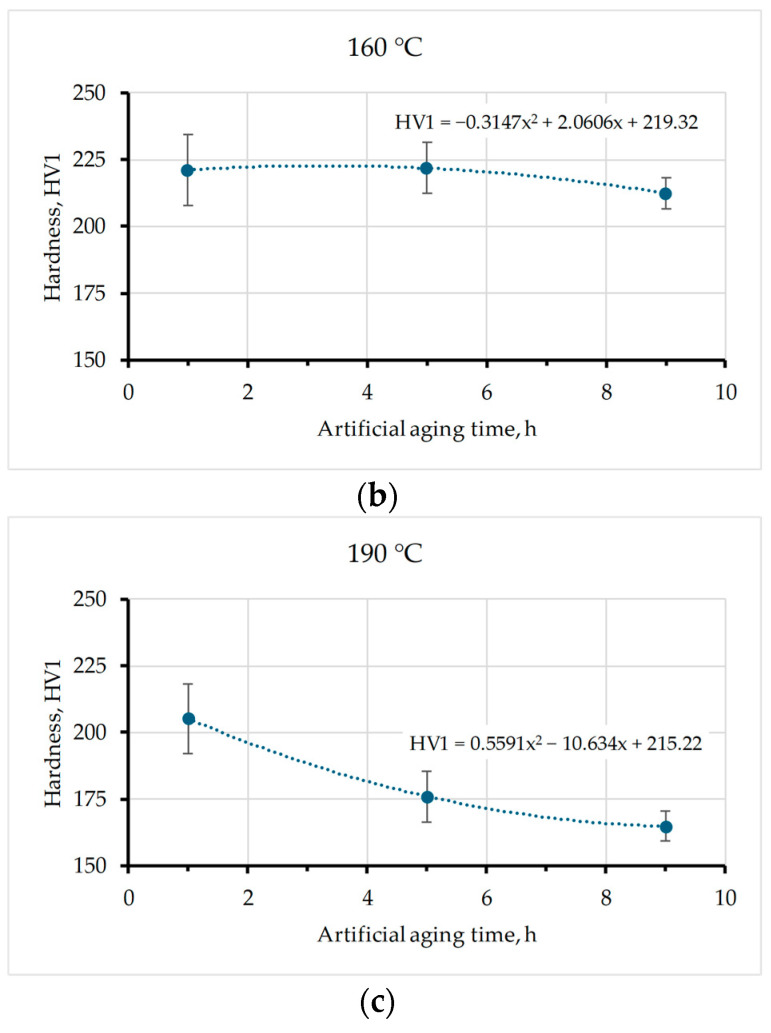
Hardness as a function of aging time, for temperatures: (**a**) 130 °C; (**b**) 160 °C; (**c**) 190 °C.

Given that wear resistance is closely related to hardness, the hardness of the samples was measured by the standard Vickers hardness method HV1 ten times for each sample. For the samples that were only solution-treated and quenched in water (ST-Q) without subsequent artificial aging, the average hardness value was 173 ± 6.21 HV1. The lowest hardness value (165 ± 9.48 HV1) was measured on the samples that underwent heat treatment at a temperature of 190 °C for 9 h, while the highest hardness value (227 ± 9.57 HV1) was measured on the samples artificially aged at a temperature of 130 °C for 5 h. The Vickers hardness, as a function of the aging time, for the temperatures of 130, 160, and 190 °C is shown in Figure 6.

**Figure 7:**
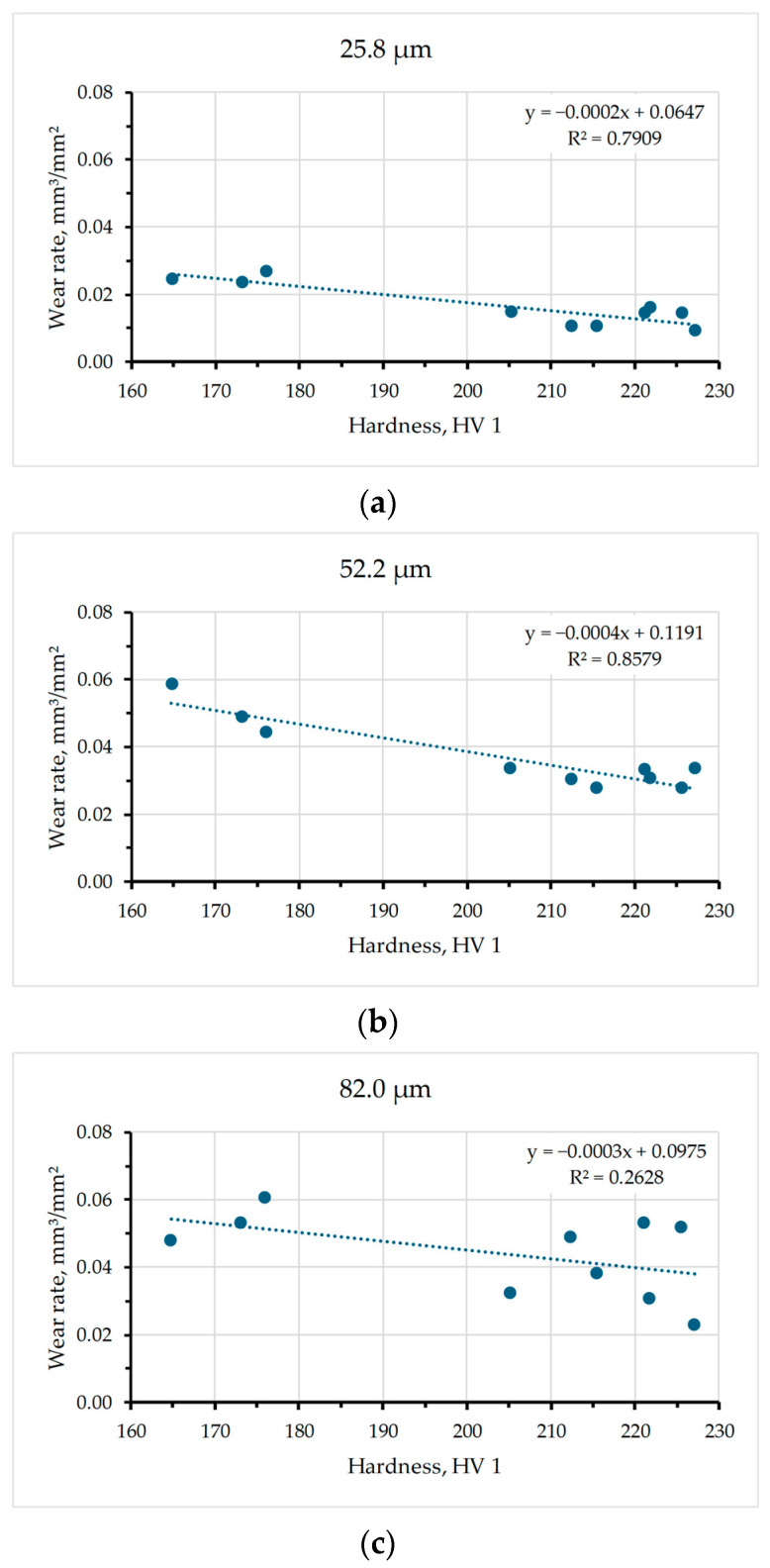
Wear rate as a function of hardness, for average abrasive grain size of (**a**) 25.8 µm (P600); (**b**) 52.2 µm (P280); (**c**) 82.0 µm (P180).

The wear rate as a linear function of hardness for the abrasive grain sizes 25.8, 52.2, and 82.0 µm which correspond to the sandpaper qualities P600, P280, and P180 is shown in Figure 7. It is visible that the abrasive grain size affects the correlation coefficient value which is very indicative. For the smallest abrasive grain size of 25.8 µm (sandpaper P600), the correlation coefficient is 0.7909, which indicates a very good correlation between the observed values, as shown in Figure 7a. When testing wear resistance by the P280 sandpaper (the abrasive grain size of 52.2 µm), the correlation coefficient is significantly larger (0.8579), which similarly indicates a very strong correlation between the wear rate and hardness, as shown in Figure 7b. For the largest abrasive grain size of 82 µm (sandpaper P180), the linear regression analysis shows a rather low correlation coefficient of 0.2628, which indicates that only 26.28% of the wear rate variation can explain the linear dependence on hardness, as shown in Figure 7c. From Figure 7, it can be observed that the slope of all three linear functions is negative, which indicates that the wear rate will decrease as the hardness value increases. The highest slope of the linear function was noticed for the abrasive grain size 52.2 µm (sandpaper quality P280), which indicates a greater change in the wear rate compared to other grain sizes. In addition, the smallest slope of the linear function was for the abrasive grain size 25.8 µm (sandpaper quality P600), which indicates the smallest rate of abrasion resistance change with varying hardness.

The combinations of factors that were used for the design of experiment method and the measured wear values are shown in Table 6. The Face-Centered central composite design (CCF) was used for the test, where the value *α* = 1 (star points are at the center of each face of the factorial space). For response 1, the mass loss (Δ*m*) after the test was weighted. For response 2, the wear rate (*ώ*), the volume loss Δ*V*, calculated as a ratio of Δ*m* and the measured density (2.7857 g/cm^3^), was divided by the cross-sectional area of the sample.

Since all three responses are related, the analysis of variance (ANOVA) and model graphs are presented for the final response only, the wear rate. For the measured and calculated results, the modified quadratic model was found to be the most suitable. Based on the ANOVA analysis, the suggested modified quadratic model of wear rate is as follows:

*ώ* = 0.152859 − 0.002328 · A + 0.000996 · B + 0.001654 · C + 0.00000743188 · A^2^ − 0.000011 · C^2^, (2)

where

- *ώ*—wear rate, mm^3^/mm^2^,
- A—aging temperature, °C;
- B—aging time, h;
- C—abrasive grain size, µm.

Other combinations of factors including AB, AC, BC, and B^2^ were not included in the model because the ANOVA pointed out that these combinations were not significant. The results of the ANOVA for this modified quadratic model of wear rate, which does not include insignificant variables, are presented in Table 7.

Since the F-value of the model is 12.25, it implies that the model is significant and there is only a 0.08% chance (*p*-value = 0.0008) that this large F-value could occur due to noise. Even though factor A has a large *p*-value, it is included in the model to support hierarchy. The coefficient of determination (R^2^) is relatively close to the value of 1, which suggests that this model describes the obtained results quite well. The predicted R^2^ of 0.6353 is in reasonable agreement with the adjusted R^2^ of 0.8008.

**Figure 8:**
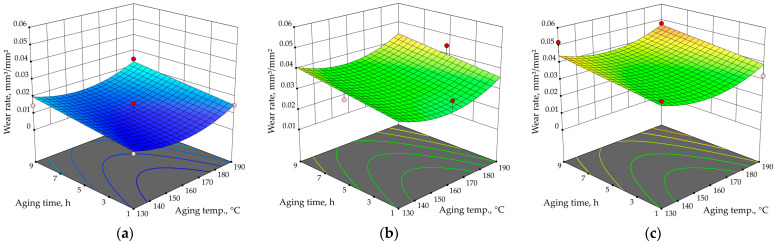
The effect of aging time and temperature on wear rate for average abrasive grain size of (**a**) 25.8 µm (P600); (**b**) 52.2 µm (P280); (**c**) 82.0 µm (P180).

**Figure 9:**
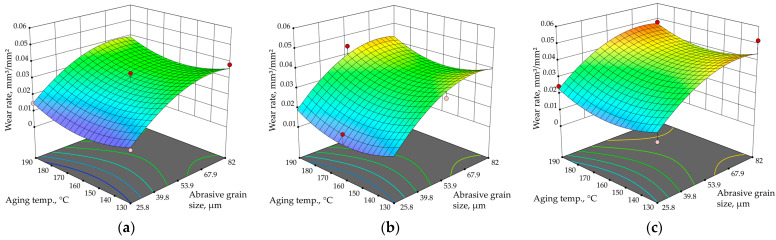
The effect of abrasive grain size and aging temperature on wear rate for aging time of (**a**) 1 h; (**b**) 5 h; (**c**) 9 h.

Figure 8 and Figure 9 show 3D graphical presentations of the combined effects of all input parameters on the wear rate of the precipitation-hardened Al-Zn-Mg alloy. Figure 8 shows the influence of aging temperature and time for various abrasive grain sizes (sandpaper qualities), while Figure 9 presents the effect of average abrasive grain size and aging temperature.

From Figure 8 and Figure 9, it is obvious that abrasive grain size has the largest influence on the wear rate of strengthened Al-Zn-Mg alloy; while using sandpaper with a larger abrasive grain size, the wear rate generally increases. On the other hand, the artificial aging time does not have a large influence on the abrasion resistance, but this may be the result of the limitation of the central composite design, as it excludes some of the experiment points. For example, artificially aged samples with the lowest and the highest wear rate (state 130/5 abraded with the finest sandpaper, *ώ* = 0.0094 mm^3^/mm^2^ and state 190/5 abraded with the roughest sandpaper, *ώ* = 0.0605 mm^3^/mm^2^, respectively) are not included in the CCF. Based solely on the results obtained from the CCF, it appears that the sample artificially aged at 130 °C for 1 h and worn with the smallest abrasive size of 25.8 µm has the lowest wear rate (*ώ* = 0.0105 mm^3^/mm^2^). This value is quite close to the actual lowest value for the examined thermal states. In addition, according to the CCF results, the highest wear rate would be for the sample artificially aged at 130 °C for 9 h and abraded with the roughest abrasive size of 82.0 µm (*ώ* = 0.0517 mm^3^/mm^2^), which is not that close to the actual maximum wear rate of 0.0605 mm^3^/mm^2^ measured for state 190/5.

## 4. Conclusions

In this study, the influence of different aging parameters (temperature and time) of Al-Zn-Mg alloy on its resistance to abrasive wear and hardness was investigated. On all the samples, solution treatment was performed at the same parameters (490 °C for 1 h with quenching in water), while the temperature and time of artificial aging were varied (130, 160, and 190 °C; 1, 5, and 9 h, respectively). Based on the results of wear resistance tests and hardness analysis, the following conclusions can be drawn:

- Artificial aging parameters (temperature and time) affect resistance to abrasion wear. It was found that aging the material at a temperature of 130 °C for 5 h induces the least wear for both the smallest (25.8 µm) and the largest abrasive grain size (82.0 µm) resulting in the lowest total wear rate (the highest wear resistance). On the other hand, the highest wear for all tested abrasive sizes, and thus the greatest total wear rate (the lowest wear resistance), was recorded when the material was artificially aged at 190 °C for 5 and 9 h.
- In some samples, there is a phenomenon known as critical abrasive grain size. This implies that after a certain abrasive grain size, the wear intensity changes, whereby the wear rate continues to increase with the increase in abrasive size, but faster or slower (130/9, ST-Q), remains approximately the same (160/5, 190/1) or even decreases (130/5, 190/9). For the samples of other thermal states (130/1, 160/1, 160/9, and 190/5), the phenomenon of critical abrasive grain size was not visible. More different abrasive particle sizes are required to accurately determine the critical abrasive grain size.
- The hardness results indicated that the Al-Zn-Mg alloy subjected to aging generally showed increased hardness. The hardness values for different thermal states ranged from 165 HV1 to 227 HV1. The sample that was artificially aged at a temperature of 190 °C for 9 h showed the lowest hardness (165 ± 9.48 HV1) which correlates with its very intense wear. On the other hand, the samples aged at 130 °C for 5 h had the highest hardness (227± 9.57 HV1) which is also consistent with the results of abrasion and the least intense total wear.
- Furthermore, it should be noted that the size of the abrasive grain highly affects the relation between wear rate and hardness.

## Figures and Tables

**Table 1:** Chemical composition of Al alloy.

Element, wt. %										
Zn	Mg	Fe	Mn	Si	Zr	Cu	Sb	Ti	Cr	Al
7.68	3.54	0.222	0.206	0.151	0.109	0.044	0.042	0.039	0.013	bal.

**Table 2:** Artificial aging temperatures and times.

State	Artificial Aging Temperature, °C	Artificial Aging Time, h
ST-Q	-/-	-/-
130/1	130	1
130/5	130	5
130/9	130	9
160/1	160	1
160/5	160	5
160/9	160	9
190/1	190	1
190/5	190	5
190/9	190	9

**Table 3:** Average abrasive grain sizes of used sandpapers.

ISO/FEPA Grit Designation	Average Abrasive Grain Size, µm
P600	25.8
P280	52.2
P180	82.0

**Table 4:** Design of experiment.

Symbol	Factor	Level		
		−1	0	+1
A	Artificial aging temperature, °C	130	160	190
B	Artificial aging time, h	1	5	9
C	Abrasive grain size, µm	25.8	53.9	82.0

**Table 5:** Mass losses and total wear rates of samples abraded with different sandpapers.

State	Mass Loss (Δ*m*), g				Total Wear Rate (*ώ*), mm^3^/mm^2^
	P600	P280	P180	Total	
ST-Q	0.0017 ± 0.0005	0.0035 ± 0.0011	0.0038 ± 0.0023	0.0090	0.1256939
130/1	0.0008 ± 0.0002	0.0021 ± 0.0005	0.0029 ± 0.0003	0.0058	0.0767269
130/5	0.0007 ± 0.0001	0.0025 ± 0.0019	0.0017 ± 0.0001	0.0049	0.0659601
130/9	0.0011 ± 0.0001	0.0021 ± 0.0007	0.0039 ± 0.0014	0.0071	0.0942676
160/1	0.0011 ± 0.0003	0.0025 ± 0.0013	0.0040 ± 0.0012	0.0076	0.1008957
160/5	0.0012 ± 0.0002	0.0023 ± 0.0008	0.0023 ± 0.0004	0.0058	0.0774652
160/9	0.0008 ± 0.0002	0.0023 ± 0.0011	0.0037 ± 0.0010	0.0068	0.0899557
190/1	0.0011 ± 0.0001	0.0025 ± 0.0014	0.0024 ± 0.0010	0.0060	0.0810531
190/5	0.0020 ± 0.0008	0.0033 ± 0.0003	0.0045 ± 0.0006	0.0098	0.1319191
190/9	0.0018 ± 0.0000	0.0043 ± 0.0002	0.0035 ± 0.0001	0.0096	0.1312447

**Table 6:** Combinations of factors and the results.

Factor A	Factor B	Factor C	Response 1	Response 2
Artificial Aging Temp., °C	Artificial Aging Time, h	Abrasive Grain Size, µm	Δ*m*, g	*ώ*, mm^3^/mm^2^
130	1	25.8	0.0008	0.0105830
130	1	82.0	0.0029	0.0383635
130	5	53.9	0.0025	0.0336531
130	9	25.8	0.0011	0.0146048
130	9	82.0	0.0039	0.0517808
160	1	53.9	0.0025	0.0331894
160	5	25.8	0.0012	0.0160273
160	5	53.9	0.0023	0.0307189
160	5	82.0	0.0023	0.0307189
160	9	53.9	0.0023	0.0304262
190	1	25.8	0.0011	0.0148597
190	1	82.0	0.0024	0.0324213
190	5	53.9	0.0033	0.0444217
190	9	25.8	0.0018	0.0246084
190	9	82.0	0.0035	0.0478496

**Table 7:** ANOVA for modified quadratic model of wear rate (SS—Sum of Squares, DF—Degrees of Freedom, and MS—Mean Square).

Source	SS	DF	MS	F-Value	*p*-Value
Model	0.0019	5	0.0004	12.25	0.0008
A: Artificial aging temperature	0.0000	1	0.0000	0.7465	0.4100
B: Artificial aging time	0.0002	1	0.0002	5.15	0.0494
C: Abrasive grain size	0.0015	1	0.0015	47.03	<0.0001
A^2^	0.0001	1	0.0001	4.06	0.0747
C^2^	0.0002	1	0.0002	7.31	0.0242
Residual	0.0003	9	0.0000		
Cor Total	0.0022	14			
Standard deviation	0.0056			R^2^	0.8719
Mean	0.0303			Adjusted R^2^	0.8008
Coefficient of variation, %	18.34			Predicted R^2^	0.6353
				Adeq. Precision	10.3284

## Data Availability

The data presented in this study are available upon request from the corresponding author.
